# Determining a cutoff score for the family burden interview schedule using three statistical methods

**DOI:** 10.1186/s12874-019-0734-8

**Published:** 2019-05-08

**Authors:** Yu Yu, Zi-Wei Liu, Wei Zhou, Mei Zhao, Bing-Wei Tang, Shui-Yuan Xiao

**Affiliations:** 1 Hospital Evaluation Office, Xiangya Hospital, Central South University, Xiangya Road 87, Changsha, 410008 Hunan China; 2 Department of Psychiatry and Mental Health Institute of the Second Xiangya Hospital, Central South University, Renmin Middle Road 139, Changsha, 410011 Hunan China; 3 Hospital Administration Institute, Xiangya Hospital, Central South University, Xiangya Road 87, Changsha, 410008 Hunan China; 4 Social medicine and health management department, Xiangya School of Public Health, Central South University, Upper Mayuanlin Road 238, Changsha, 410008 Hunan China; 5 Mental Health Center, Xiangya Hospital, Central South University, Xiangya Road 87, Changsha, 410008 Hunan China

**Keywords:** Family burden interview schedule (FBIS), Cutoff, Tree-based modeling, K-means clustering technique, Linear regression, Sensitivity, Specificity

## Abstract

**Background:**

While it is widely acknowledged that family burden can be ameliorated with effective psycho-social interventions, how to measure family burden and define a valid cutoff to identify family caregivers in need of such interventions remains a key question. The purpose of the present study was to determine a statistically valid cutoff score for the Family Burden Interview Schedule (FBIS), using the cutoff scores of the Patient Health Questionnaire (PHQ-9) and the Generalized Anxiety Disorder Scale (GAD-7) as the reference.

**Methods:**

The FBIS, PHQ-9, and GAD-7 were administered to a representative community sample of 327 family caregivers of schizophrenia patients. A FBIS cutoff score was determined using three different statistical methods: tree-based modeling, K-means clustering technique and linear regression. Contingency analysis was conducted to compare the FBIS cutoff with depression and anxiety scale scores.

**Results:**

Findings proposed a cutoff score of 23 for the FBIS, with sensitivity being 76% for PHQ-9 and 74% for GAD-7, specificity being 68% for PHQ-9 and 67% for GAD-7.

**Conclusion:**

This cutoff score would enable health care providers to assess family caregivers at risk and provide necessary interventions to improve their quality of life.

**Electronic supplementary material:**

The online version of this article (10.1186/s12874-019-0734-8) contains supplementary material, which is available to authorized users.

## Background

Caring for a family member with mental illness like schizophrenia is a laborious and time-consuming task that usually leads to adverse physical, psychological, emotional and economic impacts on family members, known as family burden [1–3].Decades of international research have established the positive correlation between family burden and a range of negative caregiver outcomes such as depression, anxiety, physical disease and even mortality [4–6]. While it is widely acknowledged that these conditions can be greatly ameliorated with effective psycho-social interventions [7–9], how to measure family burden and define a valid cutoff to identify family caregivers in need of such interventions remains a key question.

The importance of measuring family burden has long been recognized in the literature, with a large quantity of instruments developed for assessing family burden in both physical diseases such as hemanioma, atopic dermatitis, ichthyosis and mental disease such as dementia, bipolar disorder, etc. [10–13]. However, a review of past literature only detected four instruments that are specific to measuring family burden for persons with schizophrenia [14]. Among the four instruments, the Family Burden Interview Schedule (FBIS) [15] is proposed as the most promising one for its specificity, clinical application, and evidence [16].

The FBIS offered a relatively short, yet comprehensive and multidimensional assessment of family burden. Originally developed by Pai and Kapur [15] in 1981, the FBIS measures two aspects of burden (objective and subjective) encompassing six categories: financial burden, disruption of routine family activities, family leisure, family interactions, and effect on physical and mental health of others. Each category is composed of 2 to 6 items, adding up to 24 items for the whole FBIS. Each item is rated on a 3-point Likert scale from 0 (no burden) to 2 (serious burden), with a total score ranging from 0 to 48. For FBIS, the score is mostly used as a continuous variable yet no valid cutoff score has ever been proposed. However, use of continuous value for total burden score is inconvenient when it comes to deciding whether to include or not a caregiver in a burden prevention or treatment program, in this occasion, a dichotomous classification is more needed [17].

Therefore, the present study was performed to define a valid cutoff score for the FBIS to screen caregivers in need of further assessment and intervention. Considering the well-established positive association between family burden and caregiver depression and anxiety [4–6], we decided to explore FBIS cutoff score with reference to depression and anxiety score.

## Methods

### Participants and procedure

The cross-sectional study was conducted in Ningxiang County, Hunan province of China from November 2015 to January 2016. A one-stage cluster-sampling method was used to recruit family caregivers of schizophrenia patients from the 686 program, which was China’s largest demonstration project in mental health service [18], with over 3000 registered patients with serious mental illness(majority diagnosed as schizophrenia) in Ningxiang County.

A total of 55 representative communities/villages were selected from four randomly selected towns/townships from Ningxiang County. In each community/village, we recruit one primary family caregiver of schizophrenia patient from the 686 program, leading to a total sample of 352 primary family caregivers. The Inclusion criteria includes: (1) the care recipient fulfills the Chinese Classification of Mental Disorders-3 (CCMD-3) or the International Classification of Diseases-10 (ICD-10) criteria for schizophrenia; (2) the care recipient is living with at least one informal caregiver; (3) the primary caregiver is a family member of care recipient; (4) the primary caregiver is living with the patient and has taken the most responsibility of caring; (5) the primary caregiver is no less than 16 years of age; (6) the primary caregiver is able to understand and communicate.

Ethical approval was obtained from the Institutional Review Committee of the Xiangya School of Public Health of Central South University. We approached all 352 primary caregivers by door-to-door visit accompanied by the town/village doctors, who are very familiar with the caregivers and act as a guide for our home visit. After explaining the purpose of the study and obtaining written consent from the caregivers, face-to-face interviews were conducted with caregivers at their home. Among the 352 primary caregivers we approached, 14 refused to participate, 11 dropped out during the interview, leading to a response rate of 93% and 327 final respondents. Details of the study have been published elsewhere [4].

### Instruments

#### Family burden interview schedule (FBIS)

The FBIS [15] consists of 24 items asking about whether respondents have experienced burden on the following six domains: financial burden, disruption of routine family activities, family leisure, family interactions, and effect on physical and mental health of others. Answers are scored on a 3-point Likert scale from 0 = “no burden” to 2 = “serious burden” with total score ranging from 0 to 48. The Chinese version of FBIS showed acceptable internal consistency in the current study with a Cronbach’s α of 0.86.

#### Patient health questionnaire (PHQ-9)

The PHQ-9 [19] consists of 9 items asking about whether respondents have experienced 9 symptoms including the level of interest in doing things, feeling down or depressed, difficulty with sleeping, energy levels, eating habits, self-perception, ability to concentrate, speed of functioning and thoughts of suicide in the past two weeks. Answers are scored on a 4-point Likert scale from 0 = “not at all” to 3 = “nearly every day” with total score ranging from 0 to 27 and a cutoff point of 10 differentiating depression and non-depression [20]. The Chinese version of the PHQ-9 demonstrated good internal consistency in the current study with a Cronbach’s α coefficient of 0.89.

#### Generalized anxiety disorder scale (GAD-7)

The GAD-7 [21] consists of 7 items asking about whether respondents have experienced 7 symptoms including feeling nervous, cannot control worrying, worrying too much, trouble relaxing, hard to sit still, easily annoyed and feeling afraid in the past two weeks. Answers are scored on a 4-point Likert scale from 0 = “not at all” to 3 = “nearly every day” with total score ranging from 0 to 21 and a cutoff point of 10 differentiating anxiety and non-anxiety [22]. The Chinese version of the GAD-7 demonstrated good internal consistency in the current study with a Cronbach’s α coefficient of 0.91.

### Data analysis

All data were analyzed using SPSS software version 17.0. In order to identify the cutoff score for the FBIS with reference to the PHQ-9 and GAD-7, we replicated Schreiner, A. S., et al.’s [5] statistical method by utilizing the following three different statistical methods: (1) Tree-based modeling; (2) K-means clustering technique; and (3) Linear regression.

Having been proposed to be one of the best and mostly used supervised learning methods, tree based methods empower predictive models for both categorical and continuous input and output variables, and map both linear and non-linear relationships quite well [23–25]. Here we use interaction trees to capture treatment-subgroup interactions by recursively splitting the group of patients based on pretreatment characteristics, such that in each split the treatment-split interaction is maximized. In this method, we segregate the sample based on family burden score (FBIS) to predict depression as assessed by PHQ-9 and anxiety as assessed by GAD-7. Here the input variable is continuous—the FBIS score, while the output variable is categorical—depression vs non-depression, and anxiety vs non-anxiety. We chose the first decision node from the first splitting as our cutoff point, since we use dichotomy for the FBIS score.

K-means clustering is a kind of data clustering techniques to divide cases or variables of a dataset into non-overlapping groups/clusters, based on the characteristics uncovered. The goal is to produce groups of cases/variables with a high degree of similarity within each group and a low degree of similarity between groups [26–29]. In this method, we only used the FBIS score and classified the sample into high burden and low burden group by K-means clustering to get a cutoff point for the FBIS score.

For linear regression, scatterplots were firstly explored for the relationship between FBIS score with PHQ-9 and GAD-7 score, followed by both linear and non-linear relationship testing (such as quadratic terms and cubic terms) to determine a best model fit. After a linear relationship was supported, two linear regressions were performed with FBIS score as the dependent outcome variable, while PHQ-9 score and GAD-7 score as independent variables, respectively. The predicted value for the FBIS cutoff score is calculated based on the cutoff values from GAD-7 and PHQ-9 using the two linear regression models.

Using the proposed cutoff value of 10 for PHQ-9 and GAD-7 [20, 22], the samples were further grouped into high and low depression groups, high and low anxiety groups, which were compared against high and low burden groups by 2 × 2 contingency tables, respectively. Considering the increased risked of rejecting one or more true null hypotheses (i.e., of committing one or more type I errors) by multiple comparisons, we used Bonferroni correction by dividing the alpha value of 0.05 by the number of comparisons to control for type-I error. We further analyzed the sensitivity and specificity of each contingency table to test how well the FBIS cutoff as compared to the PHQ-9 and GAD-7 in assessing caregiver depression and anxiety. In the present study, sensitivity refers to the FBIS cutoff ‘s ability to correctly identify depression/anxiety subjects by the PHQ-9/GAD-7 standard while specificity means the cutoff's ability to correctly identify non-depression/non-anxiety subjects. Finally, a Youden index was calculated by the following formula: Youden index = specificity + sensitivity − 1, with a higher score representing better screening ability [30].

## Results

### Sample characteristics

Table 1 shows descriptive data on the sample. The caregiver profile corresponds to a 58-year old married, half-employed first degree relative (mostly parents or spouses), with low education and having been caring for the patients for more than 10 years. Caregiver burden was measured as 23.66 for FBIS, while the mean caregiver depression and anxiety scores were 9.75 and 9.31, respectively.

**Table 1:** Sample characteristics (*n* = 327)

Variables		*n*(%)/m(Sd)
Age		57.6(12.5)
Gender	Male	151(46.2)
	Female	176(53.8)
Marriage	Married	269(82.3)
	Unmarried	58(17.7)
Occupation	Full-employed	19(5.8)
	Half-employed	154(47.1)
	Housewife/husband	97(29.7)
	Retired	23(7.0)
	Unemployed	34(10.4)
Education	Primary(primary and below)	196(59.9)
	Middle(middle school)	87(26.6)
	High(high school and above)	44(13.5)
Kinship	Parents	151(46.2)
	Spouse	113(34.6)
	siblings	25(7.6)
	Children |	32(9.8)
	other	6(1.8)
Length of caring	< 10 yrs	84(25.7)
	≧10 yrs	243(74.3)
FBIS score		23.66(9.79)
PHQ score		9.75(7.31)
GAD score		9.31(6.61)

### Cutoff values

**Fig. 1:**
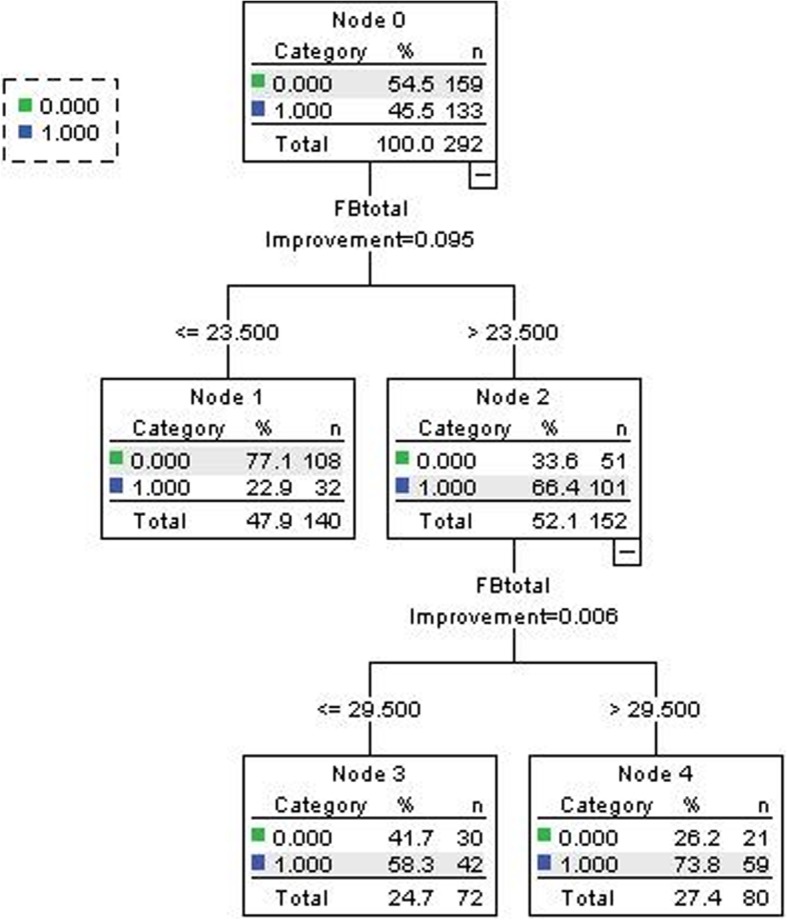
Tree-based modeling for FBIS-cutoff by PHQ-9

**Fig. 2:**
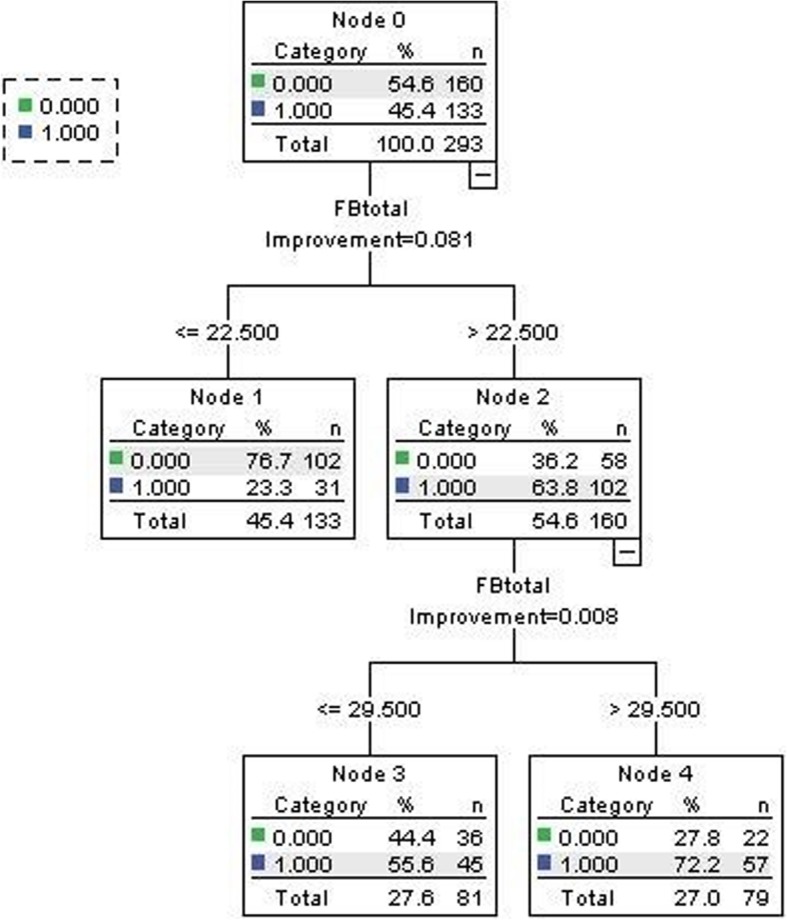
Tree-based modeling for FBIS-cutoff by GAD-7

Tree-based modeling generated a FBIS cutoff score of 23.5 for predicting depression as assessed by PHQ-9 (Fig. 1) and a FBIS cutoff score of 22.5 for predicting anxiety as assessed by GAD-7 (Fig. 2). The K-means clustering assigned a cutoff score of 23 to distinguish between high and low burden. Scatterplots between FBIS score with PHQ-9 and GAD-7 score(Additional file 1-2), as well as both linear and non-linear relationship testing supported for the linear regression model (Additional file 3-4), which suggested a cutoff score of 23.82 for PHQ-9 (cutoff set at 10) and 24.05 for GAD-7 (cutoff set at 10) (Table 2). Thus, three unique methods confirmed a FBIS cutoff around the value of 23. In an effort to search for an optimal cutoff value, we expanded our cutoff candidates by using six different burden cutoffs (20, 21, 22, 23, 24 and 25), which centered around the statistically determined cutoff of 23.

**Table 2:** Linear regression of burden scores on PHQ-9/GAD-7 scores

FBIS	Model r^2^	unstandardized coefficients			
		B	Std.Error	t	Sig.
(Constant)^a^	0.249	17.12	0.83	20.52	< 0.001
PHQ ^a^		0.67	0.07	9.8	< 0.001
(Constant)^b^	0.235	16.85	0.87	19.41	< 0.001
GAD^b^		0.72	0.08	9.45	< 0.001

Dependent variable: FBIS total burden scores

^a^ Using the PHQ-9 cutoff score of 10 results in a burden score of 23.82(17.12c + 0.67b*10 = 23.82)

^b^ Using the GAD-7 cutoff score of 10 results in a burden score of 24.05(16.85c + 0.72b*10 = 24.05)

### Contingency analysis

We further run 2 × 2 contingency analysis between six chosen candidate FBIS cutoff scores (20–25) and the cutoff scores of PHQ-9 (Table 3) and GAD-7 (Table 4). All FBIS cutoff scores were significant in predicting the risk of depression and anxiety, among which the score of 23 on FBIS showed a best Youden’s index for both PHQ and GAD. A FBIS cutoff score of 23 produced a sensitivity of 76% for PHQ and 74% for GAD, and a specificity of 68% for PHQ and 67% for GAD. The results indicated that 76% of high burden caregivers were in the probable depression group while 74% of high burden caregivers were in the probable anxiety group. In addition, 68% of low burden caregivers were with low risk of depression, while 67% of low burden caregivers were with low risk of anxiety.

**Table 3:** Contingency analysis of caregivers by depression and burden groups

Cutoff scores.	High PHQ-9	Low PHQ-9	Total N	Chi-square	Sig.^a^	Sensitivity	Specificity	Youden index
High burden> 20	112	69	181	51.198	< 0.001	84.2%	56.6%	40.8%
Low burden<=20	21	90	111					
High burden> 21	107	64	171	48.227	< 0.001	80.5%	59.7%	40.2%
Low burden<=21	26	95	121					
High burden> 22	104	57	161	52.501	< 0.001	78.2%	64.2%	42.4%
Low burden<=22	29	102	131					
High burden> 23	101	51	152	55.832	< 0.001	75.9%	67.9%	43.8%
Low burden<=23	32	108	140					
High burden> 24	96	48	144	51.09	< 0.001	72.2%	69.8%	42.0%
Low burden<=24	37	111	148					
High burden> 25	94	45	139	52.136	< 0.001	70.7%	71.7%	42.4%
Low burden<=25	39	114	153					

^a^alpha value was set at 0.004 after Bonferroni correction

**Table 4:** Contingency analysis of caregivers by anxiety and burden groups

Cutoff scores	High GAD-7	Low GAD-7	Total N	Chi-square	Sig.^a^	Sensitivity	Specificity	Youden index
High burden> 20	108	72	180	40.177	< 0.001	81.20%	55.00%	0.362
Low burden<=20	25	88	113					
High burden> 21	106	64	170	46.995	< 0.001	79.70%	60.00%	0.397
Low burden<=21	27	96	123					
High burden> 22	102	58	160	47.921	< 0.001	76.70%	63.80%	0.405
Low burden<=22	31	102	133					
High burden> 23	98	53	151	47.836	< 0.001	73.70%	66.90%	0.406
Low burden<=23	35	107	142					
High burden> 24	94	49	143	46.629	< 0.001	70.70%	69.40%	0.401
Low burden<=24	39	111	150					
High burden> 25	90	48	138	41.362	< 0.001	67.70%	70.00%	0.377
Low burden<=25	43	112	155					

^a^alpha value was set at 0.004 after Bonferroni correction

## Discussion

Schizophrenia is a debilitating, persistent psychiatric disorder that not only affects the patients who suffer from it, but also extols significant burden on family and causes psychological distress such as depression and anxiety. Family burden has been reported to be one of the major reasons for family members to give up caregiving tasks and institutionalize the patients [31, 32]. These conditions can be ameliorated with current psycho-educational interventions which focus on increasing caregivers’ knowledge and skills of patient management, alleviating caregivers’ feelings of stress, helplessness and burden, and improving caregivers’ sense of self-efficacy and self-value [33–36]. A valid FBIS cutoff score would enable health care professionals to identify family caregivers in need of such interventions to alleviate family burden and improve caregiver’s quality of life.

To our knowledge, this is the first study to determine a statistically derived cutoff score using three methods for the most commonly used FBIS scale among schizophrenia caregivers to predict both depression and anxiety. Our findings suggest a FBIS cutoff score of 23 to identify caregivers at risk of both depression and anxiety and thus in need of further assessment and intervention. It has a positive predictive value of 76% for PHQ-9 and 74% for GAD-7, which indicates that 76% or 74% of caregivers above the FBIS cutoff are also above the depression cutoff or the anxiety cutoff. The negative predictive value is 68% for PHQ-9 and 67% for GAD-7, implying that 68% or 67% of caregivers below the FBIS cutoff are also below the depression cutoff or the anxiety cutoff.

The findings also imply some added benefits for the use of the FBIS scale by indicating that it not only measures family burden, but also assess the extent to which family burden constitutes psychological distress such as depression and anxiety for caregivers. In other words, caregivers at risk for depression and anxiety may be identified by administering the FBIS alone. In addition, the FBIS has been mostly used as a continuous variable with no cutoff point proposed in the past, the finding of the current study may fill in the research gap of lacking a FBIS cutoff value to distinguish families who are in need of further intervention from those who are not simply by FBIS score. Future family intervention program targeted at alleviating family burden and improving caregiver well-being may benefit from this cutoff as selection criterion.

In this study, we used three different analytical methods to determine a cutoff value for the FBIS score. Although they are different in definition, scope of application, terminologies, and analytic codes, they produce basically similar cutoff values for the FBIS score, implying the wide applicability and robustness of the three methods. However, cautions also need to be paid during the choice of each method. For tree-based modeling, although it has the advantage of being easy to understand, being useful in data exploration, requiring less data cleaning, with no constraint on data type, and being non-parametric method, it still confronts with the challenge of over fitting, which is one of the most practical difficulties for decision tree models and can only be solved by setting constraints on model parameters and pruning [23–25]. For k-means clustering, its ease of implementation, computational efficiency and low memory consumption has kept it very popular, yet its sensitivity to the initial centroids chosen, the potential bias to create clusters of equal size, and lack of robustness to outliers require further adjustment while using this method [29, 37]. Linear regression is the first type of regression analysis to be studied rigorously and used extensively in practical applications. However, it makes a number of assumptions about the predictor variables, the response variables and their relationship. These assumptions include weak exogeneity, linearity, constant variance, independence and lack of perfect multicollinearity. Violations of these assumptions may need various extensions based on this model to allow relaxation [38, 39].

The study falls short in the following aspects. First of all, we used multiple statistical methods to run greedy cutoff searching, which may lead to inflated type I error. However, we re-run our analyses by splitting our sample into training set and validation set first by 1:1, then by 7:3, and found little difference of results. Considering the much smaller sample size of the split sample and related lower statistical power, we only displayed the results for the total sample testing. Future research may consider using a much larger sample size and randomly splitting it into training set and validation set with more power. Another limitation is the use of brief screening scales such as PHQ-9 and GAD-7 to assess depression and anxiety, instead of standard psychometric scales such as the Beck Depression Inventory (BDI), the Beck Anxiety Inventory (BAI), the Hamilton Rating Scale for Depression (HRSD), or the Hamilton Rating Scale for Anxiety (HRSA), which may compromise the accuracy of our measurement and thus leading to bias. However, the aim of the present study was to determine a cutoff score for the FBIS using depression and anxiety as a reference rather than to accurately measure these concepts, the results may not be affected by the choice of measurement tools. Future study may consider using standard psychometric scales and test whether brief screening scales are comparable to them. Thirdly, the use of one single cutoff value for the FBIS may introduce some kind of bias by treating persons with an FBIS score of 1 and a score of 22 as “equal” since they are both under the cutoff threshold, which is a major limitation for dichotomizing continuous variables for all scales. However, the aim of the current study was not to distinguish between various level of family burden, but to screen for those with higher burden and thus at risk for depression and anxiety for further intervention, which can be satisfied by having a cutoff value for the FBIS. Future studies focused on differentiating various levels of family burden may consider classifying the FBIS score into several levels instead of two. Also, the results of the current study are intended to serve only as a guideline for practitioners to assess their family caregivers and encourage them for further assessment and future intervention. In addition, the cutoff scores in this study warrant further test and validation in caregivers of other mental disorders.

## Conclusion

In short, the present study proposes a statistically derived cutoff score for the FBIS among caregivers of schizophrenia patients in a Chinese rural community, which may also be tested and used among other populations in other countries. The findings suggest a FBIS cutoff score of 23 has the best predictive validity for identifying caregivers at risk for depression and anxiety for further assessment and future intervention.

## Additional files

Additional file 1: **Figure S1** Scatterplots of the relationship between FBIS score with PHQ-9 score (DOCX 41 kb)

Additional file 2: **Figure S2** Scatterplots of the relationship between FBIS score with GAD-7 score (DOCX 43 kb)

Additional file 3: **Table S1** Model summary and parameter estimates of the relationship between FBIS score with PHQ-9 score (DOCX 40 kb)

Additional file 4: **Table S2** Model summary and parameter estimates of the relationship between FBIS score with GAD-7 score (DOCX 39 kb)

## Abbreviations

BAI: Beck Anxiety Inventory
BDI: Beck Depression Inventory
CCMD-3: Chinese Classification of Mental Disorders-3
FBIS: Family Burden Interview Schedule
GAD-7: Generalized Anxiety Disorder Scale
HRSA: Hamilton Rating Scale for Anxiety
HRSD: Hamilton Rating Scale for Depression
ICD-10: International Classification of Diseases-10
PHQ-9: Patient Health Questionnaire
