# Supplementary figures and images for: Determining a cutoff score for the family burden interview schedule using three statistical methods
Source: BMC Med Res Methodol. 2019 May 8;19:93. doi: 10.1186/s12874-019-0734-8 (PMC6505248; doi:10.1186/s12874-019-0734-8)

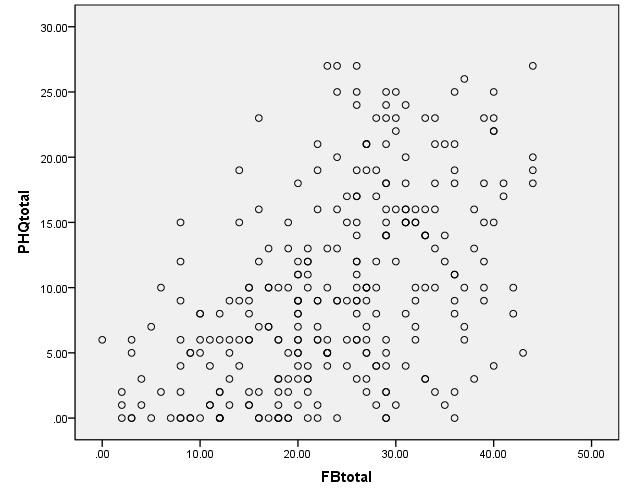


Figure1. Scatterplots of the relationship between FBIS score with PHQ-9 score

Supplement: Supplementary file 1 — Figure S1 Scatterplots of the relationship between FBIS score with PHQ-9 score (DOCX 41 kb) [file 12874_2019_734_MOESM1_ESM.docx]

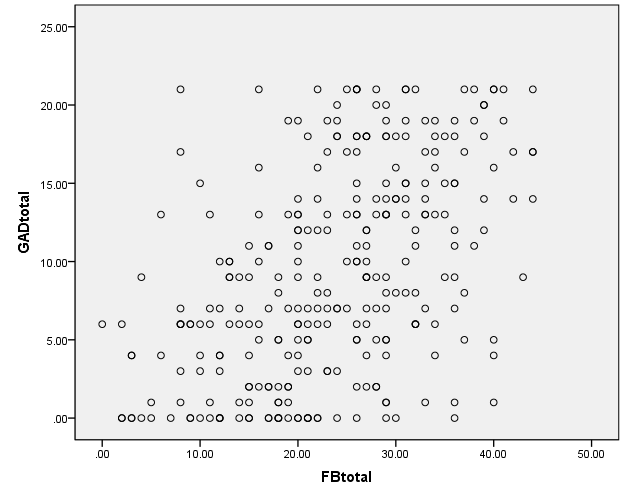


Figure 2. Scatterplots of the relationship between FBIS score with GAD-7 score

Supplement: Supplementary file 2 — Figure S2 Scatterplots of the relationship between FBIS score with GAD-7 score (DOCX 43 kb) [file 12874_2019_734_MOESM2_ESM.docx]
