# Supplementary material for: Determining a cutoff score for the family burden interview schedule using three statistical methods
Source: BMC Med Res Methodol. 2019 May 8;19:93. doi: 10.1186/s12874-019-0734-8 (PMC6505248; doi:10.1186/s12874-019-0734-8)

Table 1. Model summary and parameter estimates of the relationship between FBIS score with PHQ-9 score


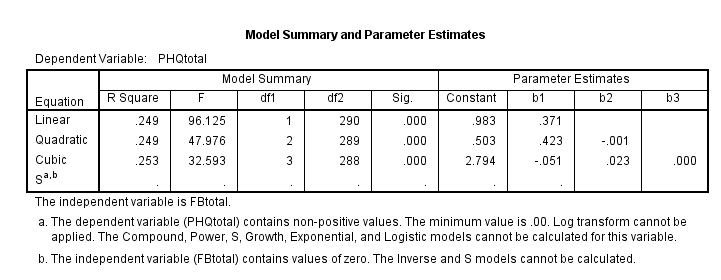

Supplement: Supplementary file 3 — Table S1 Model summary and parameter estimates of the relationship between FBIS score with PHQ-9 score (DOCX 40 kb) [file 12874_2019_734_MOESM3_ESM.docx]
