# Supplementary material for: Determining a cutoff score for the family burden interview schedule using three statistical methods
Source: BMC Med Res Methodol. 2019 May 8;19:93. doi: 10.1186/s12874-019-0734-8 (PMC6505248; doi:10.1186/s12874-019-0734-8)

Table 2. Model summary and parameter estimates of the relationship between FBIS score with GAD-7 score


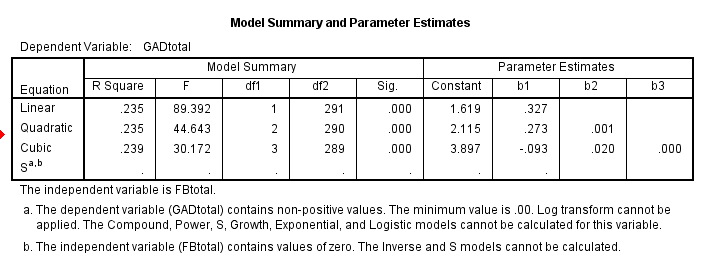

Supplement: Supplementary file 4 — Table S2 Model summary and parameter estimates of the relationship between FBIS score with GAD-7 score (DOCX 39 kb) [file 12874_2019_734_MOESM4_ESM.docx]
